# Neural tracking of continuous speech reveals enhanced late responses to degraded speech

**DOI:** 10.3389/fnins.2026.1751421

**Published:** 2026-02-06

**Authors:** Youngmin Na, Luong Do Anh Quan, Hyosung Joo, Inyong Choi, Jihwan Woo

**Affiliations:** 1 Department of Biomedical Engineering, University of Ulsan, Ulsan, Republic of Korea; 2 Department of Electrical, Electronic and Computer Engineering, University of Ulsan, Ulsan, Republic of Korea; 3 Department of Communication Sciences and Disorders, University of Iowa, Iowa City, IA, United States

**Keywords:** compensatory neural processing, degraded speech, neural tracking, speech perception, temporal response functions

## Abstract

**Introduction:**

Comprehending degraded speech demands greater cognitive effort. While previous studies have identified the neural pathways involved in processing degraded speech signals, the temporal dynamics of these neural networks remain unclear.

**Methods:**

This study investigated the time course of neural responses to clean and degraded (i.e., noise-vocoded) speech signals using temporal response functions (TRFs).

**Results:**

Our findings reveal that early TRF components (N1TRF and P2TRF) exhibited greater amplitude and temporal precision for clean speech. In contrast, degraded speech elicited additional cortical responses with a longer delay, designated as P400TRF. Subsequent source localization analyses showed that the P400TRF component originates from language processing areas within the temporal and frontal lobes.

**Discussion:**

These findings highlight the role of delayed neural mechanisms in maintaining speech comprehension when intelligibility is compromised, offering novel insights that broaden our understanding of auditory cortical processing under challenging listening conditions.

## 1 Introduction

When speech signals become noisy, distorted, or challenging to comprehend, the human auditory system compensates by combining high-level cognitive processes and low-level auditory mechanisms. The brain engages a complex network of neural systems to process degraded speech, integrating sensory, cognitive, and linguistic brain regions in a coordinated manner (Obleser and Kotz, 2010; Karunathilake et al., 2023b). Additionally, studies have highlighted that phase-locked responses in the auditory cortex are enhanced during speech comprehension, underscoring the role of neural synchronization in processing complex auditory information (Peelle et al., 2013; Hauswald et al., 2022; Kösem et al., 2023).

In the context of degraded speech, auditory cortical areas, such as the primary auditory cortex (A1) and secondary auditory regions, are primarily responsible for processing the degraded acoustic features. These regions encode basic elements like pitch, duration, and spectral properties, even when noise or reverberation is present. However, when speech intelligibility is compromised—due to background noise or low-quality signals—the brain recruits higher-level cortical regions, particularly in the prefrontal cortex and parietal areas, which involve attentional control, working memory, and top-down modulation. These regions enable the brain to fill perceptual gaps, stabilize auditory representations, and improve the listener’s ability to extract meaningful speech information (Davis and Johnsrude, 2007; Obleser and Kotz, 2010; Karunathilake et al., 2023b). Moreover, robust cortical entrainment to the speech envelope, which relies on the spectro-temporal fine structure, plays a critical role in maintaining speech intelligibility in challenging environments (Ding et al., 2014; Hauswald et al., 2022).

The neural mechanisms extend beyond simple auditory processing by integrating semantic and linguistic expectations to enhance speech intelligibility. For instance, the left inferior frontal gyrus (IFG), associated with syntactic and semantic processing, has been shown to become more active when listeners are attempting to make sense of speech under challenging conditions (Obleser et al., 2007; Peelle et al., 2010; Peelle and Wingfield, 2022). Additionally, the cortical entrainment response is significantly influenced by prior knowledge and speech intelligibility, indicating that listeners’ expectations and contextual understanding are vital for effective neural tracking of speech (Baltzell et al., 2017; Karunathilake et al., 2023b).

While temporal response function (TRF) studies have predominantly used extended speech stimuli, recent research supports the efficacy of shorter sentences (Vanthornhout et al., 2019; Das et al., 2020; Muncke et al., 2022; Slaats et al., 2023; Pendyala et al., 2024). Short sentences have been shown to provide reliable neural tracking responses and are effective in studying speech intelligibility (Muncke et al., 2022; Slaats et al., 2023).

Nevertheless, an important aspect that remains underexplored is how the timing of neural responses contributes to the comprehension of degraded speech. Most previous studies investigating the neural processing of degraded speech have primarily focused on the spatial localization of neural activity, which identifies which brain regions are activated in response to degraded speech. However, neural timing plays a crucial role in speech perception, as it determines how efficiently the brain processes degraded signals over time. Examining the onset, duration, and temporal patterns of neural responses can offer valuable insights into how the brain adapts to distorted auditory input. Understanding these time-varying dynamics is key to revealing how auditory signals are integrated and compensated for, particularly in noisy or unclear conditions.

Important findings into the time course of speech perception have emerged from eye-tracking experiments using the Visual World Paradigm (VWP; Huettig et al., 2011). VWP posits that language is processed incrementally. In spoken words, the initial portions of a speech signal often do not provide enough information to immediately identify the word’s meaning. This initial uncertainty leads to temporary but significant ambiguity among several possible words, resulting in a process known as lexical competition. Multiple potential word candidates are activated during this process and compete for selection. Lexical competition is critical in real-time language processing and is crucial for understanding how listeners decipher speech in everyday environments. Even when a word is clearly pronounced, listeners with normal hearing still experience temporary competition among these possible lexical candidates. The mechanisms through which typical listeners resolve these ambiguities have been extensively studied (e.g., McQueen et al., 1999; Dahan and Magnuson, 2006).

Immediate competition has been observed across various groups, including infants (Swingley et al., 1999; Fernald et al., 2001), adolescents (Sekerina and Brooks, 2007; Rigler et al., 2015), and postlingually deafened adults using cochlear implants (CIs) (Farris-Trimble et al., 2014). However, CI users face unique challenges due to the degraded auditory signal provided by these devices (Wilson and Dorman, 2008). These challenges highlight the differences in how CI users, especially prelingually deaf children, might process spoken language compared to typical listeners. In particular, prelingually deaf children using CIs may experience word recognition processes that differ significantly from typical listeners (McMurray et al., 2017). Despite research on lexical competition across various groups, there is a notable lack of studies investigating the neural mechanisms underlying these processes, especially in cochlear implant users. Addressing this gap is crucial, as understanding the neural basis of these strategies could lead to improvements in CI technology and rehabilitation methods. In recent years, several groups have advanced our understanding of neural responses in cochlear implant users using electrophysiological and neuroimaging approaches (Dimitrijevic et al., 2019; Langner et al., 2021; Prince et al., 2021). These studies provide important context for interpreting neural data in CI users and underscore the relevance of investigating neural processing alongside behavioral outcomes.

Recent auditory neuroscience frameworks suggest that predictive coding may provide a comprehensive explanation for how the brain processes degraded speech (Rao and Ballard, 1999; Friston, 2005). Predictive coding proposes that the brain continuously generates predictions about upcoming sensory input and minimizes the difference between expected and actual input through a process of error correction (Sohoglu and Davis, 2016; Heilbron et al., 2022). In degraded listening conditions, listeners may rely more heavily on top-down predictions to compensate for the reduced acoustic detail. Importantly, predictive coding mechanisms can operate even during passive listening, supporting the idea that linguistic and auditory processing continues automatically without requiring active attention (Todorovic et al., 2011; Auksztulewicz and Friston, 2016; Sedley et al., 2016; Sohoglu and Davis, 2016). This framework aligns with the observed neural tracking patterns in both early and late time windows under degraded speech conditions, offering a strong theoretical basis for understanding speech processing when auditory input is compromised.

Understanding the temporal dynamics of speech perception in CI users is crucial for developing more effective auditory prostheses and improving communication abilities (Sharma et al., 2002b). To address this gap, it is necessary to delve into how the brain responds to various types of speech input, particularly under conditions that replicate the auditory experiences of CI users. Investigating neural responses to different forms of lexical competition in listeners requires analyzing their reactions to continuous speech. In response to speech features, the brain demonstrates neural tracking during language processing (Lalor and Foxe, 2010). This neural tracking can provide insights into how different populations process speech, especially when the auditory signal is less than optimal.

Studies have used speech envelopes to measure neural responses to continuous speech, as these envelopes capture essential temporal dynamics of speech that correlate with EEG signals (e.g., Ding and Simon, 2012a; Di Liberto et al., 2015; O’Sullivan et al., 2015). Recent research showed that older adults exhibit stronger tracking of speech envelopes even in conditions with poorer signal-to-noise ratios (Decruy et al., 2021; Karunathilake et al., 2023a), possibly due to compensatory mechanisms to maintain comprehension (Presacco et al., 2016). These findings suggest that the brain may adapt to degraded auditory conditions by enhancing certain neural processes to maintain speech understanding. This enhanced neural tracking likely reflects the increased cognitive effort required to resolve lexical competition in challenging listening conditions. Additionally, neural tracking has been studied to indicate how the brain processes new linguistic information, particularly in high lexical competition scenarios. Such studies often reveal specific neural signatures, such as the N400 event-related potential, which reflect the cognitive effort involved in integrating challenging or unexpected words within a sentence (Broderick et al., 2018; Koskinen et al., 2020; Weissbart et al., 2020; Toffolo et al., 2022). These late neural tracking responses are typically delayed relative to word onset and are associated with higher cognitive effort required for semantic integration, especially under conditions of high lexical competition (Lau et al., 2008; reviews on N400 responses: Kutas and Federmeier, 2011; Frank et al., 2015; Frank and Willems, 2017).

Researchers often use noise-vocoded speech to replicate the auditory experience of cochlear implant users (Friesen et al., 2001; Rosen et al., 2013). This method allows researchers to simulate the auditory challenges faced by CI users and study how these challenges affect language processing at the neural level. In this study, we employed EEG recordings and speech envelope tracking techniques while presenting participants with both clear and degraded speech sentences. We aim to understand better how degraded auditory input impacts lexical processing by investigating the neural dynamics within the predictive coding framework. We specifically test whether degraded speech primarily elicits increased reliance on top-down predictions and enhanced error correction mechanisms that facilitate speech processing in challenging listening conditions. The findings from this study could contribute to developing more effective and targeted auditory training and rehabilitation strategies for CI users.

Here, we compare the TRF differences between two speech intelligibility conditions. Using scalp EEG, within-subject comparisons between cortical neural tracking of highly intelligible natural sentences and barely intelligible vocoded sentences were performed by extracting temporal envelope-related responses. In the early time range (approximately 100 ms after sound onset), the N100 component (N1), typically associated with early auditory processing and sensory abstraction stages, is expected to show stronger responses in the natural sentences condition (Näätänen, 2001; Krumbholz et al., 2003; Obleser et al., 2006). Similarly, the P2 component (approximately 200 ms after sound onset), which follows the N1 and reflects higher-level auditory processing including attention and stimulus evaluation, is also anticipated to exhibit enhanced responses in the natural speech condition. Although traditionally linked with early sensory processing, stronger N1 and P2 responses in the natural sentence condition could indicate more efficient or immediate engagement with the speech signal, facilitating quicker lexical competition and selection (Commuri et al., 2023).

Moreover, we anticipate stronger late responses in the vocoded sentences condition for the typical trajectory of semantic integration effort (N400, occurring around 300–500 ms after sentence onset). Recent studies employing TRF analyses have revealed not only early components such as N1 and P2, but also prominent later responses (often termed “P400” TRFs) that are thought to parallel the classic N400 ERP associated with semantic processing (e.g., Broderick et al., 2018). We hypothesized that degraded speech would elicit increased reliance on top-down predictions and enhanced error correction mechanisms, reflected in delayed and enhanced late neural responses, as the brain works to resolve increased prediction error (Auksztulewicz and Friston, 2016; Sohoglu and Davis, 2016; Heilbron and Chait, 2018).

We first tested the above hypothesis using sensor-space signals from central electrodes (i.e., FCz, Cz, and CPz) that are commonly employed to investigate auditory cortex activities related to speech perception (Phillips et al., 2000; Näätänen, 2001; Čeponien et al., 2002; Tremblay et al., 2003; Martin et al., 2008; Brandmeyer et al., 2013; Kayser et al., 2015; Jafarpisheh et al., 2016; Khalighinejad et al., 2017; Steinmetzger and Rosen, 2017; Drennan and Lalor, 2019; Etard and Reichenbach, 2019; Mai and Wang, 2019; Synigal et al., 2020). Subsequently, we further tested the same hypothesis in source space within six bilateral cortical regions of interest (ROI) that were selected based on previous studies that demonstrated the involvement of bilateral Heschl’s gyrus (HG), the planum polare (PP), the planum temporale (PT), the supramarginal gyrus (SMG), inferior frontal gyrus (IFG), and the middle temporal gyrus (MTG).

## 2 Materials and methods

### 2.1 Participants

Fifty normal-hearing subjects aged between 20 and 33 (mean, 24.1 years; standard deviation, 2.4; 25 men and 25 women) participated in this study. All study procedures were reviewed and approved by the Institutional Review Board of the University of Ulsan. All participants signed an informed consent form, and the study was carried out under approved guidelines.

### 2.2 Behavioral speech intelligibility (SI) test and stimuli

The 4-channel vocoded and natural conditions were employed. The Korean Sentence Recognition Test for adults was conducted to obtain the behavioral SI scores prior to EEG data acquisition (Jang et al., 2008). A 4-channel vocoder degraded the sentences to provide a lower speech intelligibility condition, as Wilson et al. (1991) outlined. The vocoder implementation used a logarithmically-spaced filter bank spanning 200–5,000 Hz. The envelope of each frequency band was extracted using a 200 Hz low-pass filter and used to modulate Gaussian white noise carriers, which were then synthesized sequentially to reconstruct the speech signal (Mehta and Oxenham, 2017). This process preserved temporal envelope cues while removing spectral fine structure, resulting in moderate spectral degradation characteristic of 4-channel vocoded speech. The SI scores of the 4-channel vocoded conditions were significantly lower than those in the natural speech condition (*p* < 0.05; paired *t*-test, Table 1). Ten continuous Korean sentences, each lasting less than 2.5 s, were selected from the Korean Standard Sentence Lists for Adults (KS-SL-A) (Jang et al., 2008). To avoid stimulus overlap between behavioral and EEG testing, the behavioral SI test employed 10 sentence sets that did not contain any of the sentences selected for the EEG experiment.

**Table 1:** Means and standard deviations for word-recognition scores for both speech intelligibility conditions.

Speech conditions	Word-recognition score (%)		*p*-value
	Mean	SD	
4-channels vocoded	78.6	12.56	*
Natural	99.65	1.01	

*p < 0.05, SD, standard deviation.

*p*-values were obtained from the paired *t*-test between the natural and 4-channels vocoded condition.

### 2.3 EEG recording and processing

Participants were seated approximately 1 m apart from two loudspeakers while watching a silent movie. Sentences were randomly played at 65 dB SPL through the loudspeakers in a soundproof room under two conditions: passive listening to vocoded speech (degraded condition) and natural speech (clean condition). This presentation level was selected to ensure comfortable listening while maintaining consistent audibility across all participants. To minimize training effects and ensure that participants’ performance in the degraded condition was not influenced by prior exposure to clear speech, all participants first completed the degraded condition, followed by the clean condition. Critically, the identical set of 10 sentences was used in both the vocoded and natural conditions. Each sentence was repeated 100 times with an inter-stimulus interval of 3 s in a randomized order within each condition block. This design allowed for direct comparison of neural responses to the same linguistic content under different acoustic intelligibility conditions. Brain activity during the passive listening tasks was recorded using a 64-channel EEG system (Biosemi Active 2 system, Biosemi Co., Netherlands) at a sampling rate of 2,048 Hz. The EEG data were band-pass filtered between 1 and 57 Hz using a finite impulse response filter. Signals were subsequently downsampled to 256 Hz and segmented into epochs of 3 s, starting 0.5 s before stimulus onset. The estimated potential was computed by averaging 100 epochs and filtered through a 1–15 Hz band-pass filter for further analysis.

### 2.4 Control analysis: alpha power as a marker of alertness

To address potential confounding effects of block order on alertness and fatigue, we conducted a control analysis examining alpha power (8–12 Hz), a well-established electrophysiological marker of vigilance and cognitive engagement (Pivik and Harman, 1995; Lai et al., 2022). Alpha band activity (8–12 Hz) was extracted from the preprocessed EEG and averaged across parietal sites (Pz, POz). Mean alpha power was compared between vocoded and natural condition using paired *t*-tests (two-tailed, degrees of freedom = 49, *n* = 50). The absence of significant differences would indicate that condition effects are not confounded by fatigue/ alertness changes.

### 2.5 TRF estimation

An auditory spectrogram was created, containing 128 spectrally resolved sub-band envelopes of the speech signals, using the NSL toolbox (Chi et al., 2005). These sub-bands are logarithmically spaced between approximately 90 and 4,000 Hz. The speech envelopes were obtained by averaging all sub-band envelopes across frequency bands. The speech envelopes were downsampled to a sampling rate of 256 Hz to match the sampling rate of the EEG signals. Baseline normalization was performed by subtracting the baseline mean from the EEG signals and then dividing by the baseline standard deviation. The baseline period (0.5 s before stimulus onset) was pooled across epochs and channels to estimate the baseline mean and standard deviation. This normalization was applied across epochs and channels to ensure consistency across epochs and preserve the relative power differences across channels.

The TRFs of natural and vocoded condition were estimated using the multivariate temporal response function (mTRF) Toolbox (Ding and Simon, 2012b; Crosse et al., 2016). The time window ranged from −100 to 600 ms. The ridge parameter (*λ*), which control for overfitting, was searched over a range from 10^−6^ to 10^6^. For each λ, TRF model performance was validated using a *leave-one-out* cross-validation (across epochs) approach, with performance quantified by the mean squared error (MSE) (averaged across channels) on unseen data (Browne, 2000). Model performance for each condition at each λ was then averaged over subjects. The λ value that yielded the lowest MSE for both conditions was 750 and was subsequently used as the optimal ridge parameter. Finally, a grand average of TRFs was obtained for each condition.

The global field power (GFP) of TRFs was computed for each participant in both the natural and 4-channel vocoded speech conditions. For sensor-level analysis, paired *t*-tests (two-tailed, degrees of freedom = 49, *n* = 50) were performed at each time point to compare GFP between conditions. The resulting *p*-values were corrected for multiple comparisons using the false discovery rate (FDR) method (Benjamini and Hochberg, 1995). Three continuous clusters of significant time periods (80–103 ms, 131–185 ms, and 376–408 ms) were identified, corresponding to the N1_TRF_, P2_TRF_, and P400_TRF_ components, respectively, following conventional auditory evoked potential nomenclature. For each period, *t*-values comparing natural and vocoded conditions are shown in the figures using color coding. Additionally, GFPs for each condition were compared with their respective baselines (average amplitude from −100 ms to 0 ms) to identify significant response periods (two-tailed paired t-test, degrees of freedom = 49, *n* = 50).

### 2.6 TRF components validation: permutation testing

To validate that identified TRF components (N1, P2, and P400) represent genuine stimulus–response coupling rather than chance-level noise or artifacts, permutation testing was conducted. Stimulus envelopes were shuffled relative to EEG data to generate a null distribution of TRF amplitudes expected under the assumption of no true stimulus–response relationship. For each subject and condition (vocoded and natural), TRFs were computed using the identical analysis pipeline as the original analysis, except stimulus envelopes were shuffled relative to the EEG recordings. Valid TRFs (computed from original, non-shuffled stimulus-EEG pairings) were compared against this null distribution (shuffled TRFs) at each time point and each condition. Peak amplitudes of TRF components (N1, P2, and P400) were compared between valid TRFs and shuffled TRFs using paired *t*-tests (two-tailed, *α* = 0.05, with FDR correction for multiple comparisons). Significantly higher valid TRF amplitudes would demonstrate that components reflect genuine neural tracking of the speech signal rather than noise.

### 2.7 TRF at source level using the inverse problem

The inverse problem was solved to calculate the source level of the evoked potentials using the MNE-python toolbox with 64-electrode EEG signals at the sensor level. First, the linear inverse operator was assembled using the noise covariance computed from the baseline period of each epoch (−500 ms to 0 ms) and the forward operators provided by FreeSurfer (Fischl, 2012). Then, the inverse operator was used to project EEG signals to 20,484 voxels at the source level using dSPM noise normalization (Dale et al., 2000). Only the orientation perpendicular to the cortical surface was extracted to compute the TRF at the source level.

The TRF of the source level was generated using the mTRF toolbox between the source current intensity and the speech temporal envelope at the same time points corresponding to the significant periods identified at the sensor level (N1_TRF_, P2_TRF_, and P400_TRF_) for each voxel. The ridge parameter *λ* was set to 750, the same as the sensor level. For source-level analysis, paired *t*-tests (two-tailed, degrees of freedom = 49, *n* = 50) were performed at each of 20,484 voxels comparing natural and 4-channel vocoded conditions, with statistical significance set at *p* < 0.05 and FDR correction applied across all voxels. The *t*-values are denoted by colors in the figures. Regions of interest were defined using the Destrieux atlas implemented in FreeSurfer (Fischl, 2012). Six bilateral ROIs were selected based on their established roles in speech processing: HG, PP, TP, SMG, MTG, and IFG. These regions were chosen to capture both early auditory processing and higher-level language areas.

## 3 Results

### 3.1 TRF at sensor level

**Figure 1:**
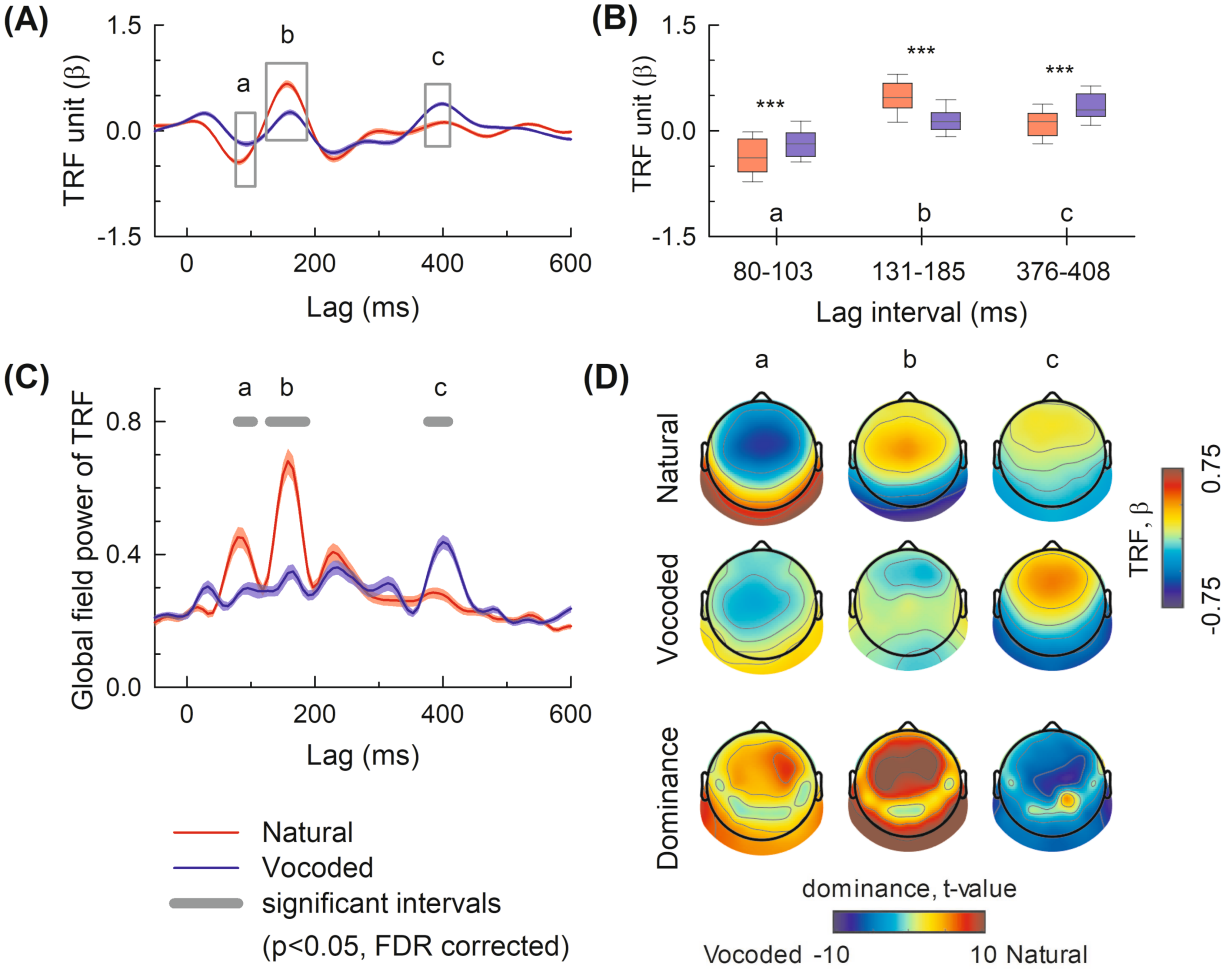
**(A)** Grand averaged TRF at the central electrodes (i.e., FCz, Cz, CPz). The black boxes represent significantly different periods between the natural (red line) and vocoded (blue line) conditions. The solid line denotes the grand average TRF, while the shaded area denotes the standard error of TRFs in each condition. **(B)** The box plot represents TRF amplitude at the N1_TRF_ (80–103 ms), P2_TRF_ (131–185 ms), and P400_TRF_ (376–408 ms). **(C)** The global field power (GFP) of TRF is in the natural (red) and vocoded (blue) condition. **(D)** Topography of TRF components in the natural (top), in the vocoded (middle), and dominance (bottom) at the N1TRF, P2TRF, and P400TRF time lags. Topographic dominance maps show the results of paired *t*-tests comparing natural vs. vocoded conditions at each electrode (*t*_49_, *p* < 0.05, FDR-corrected). Red indicates significantly larger responses to natural speech, blue indicates significantly larger responses to vocoded speech. The *t*-value scale ranges from −10 to +10 to accommodate the full range of statistical differences observed across electrodes. The central area significantly differs in the N1TRF, P2_TRF_, and P400_TRF_ components.

Figure 1A displays the grand average TRFs at central electrodes (FCz, Cz, CPz) for both conditions. Figure 1C shows the corresponding GFP, which quantifies the overall neural response strength across all electrodes. The mTRF analysis revealed distinct components: N1_TRF_, P2_TRF_, and P400_TRF_ (Figure 1A). GFP analysis identified three time periods with statistically significant differences between conditions (FDR-corrected paired *t*-tests, *t*(49), *p* < 0.05): 80–103 ms (N1_TRF_), 131–185 ms (P2_TRF_), and 376–408 ms (P400_TRF_), respectively. Notably, significant differences were observed between the natural and vocoded conditions at the central electrodes for N1_TRF_ (80–103 ms) and P2_TRF_ (131–185 ms), with larger amplitudes in the natural condition (*p* < 0.05, paired t-test, FDR-corrected) (Figure 1B). Additionally, the P400_TRF_ within 376,408 ms showed more extensive responses in the vocoded condition than in the natural condition (*p* < 0.05, paired t-test, FDR-corrected) (Figure 1B).

The topographic maps of grand average TRF for the natural and vocoded conditions (first and second row) and their *t*-value (third row) at the N1_TRF_, P2_TRF_, and P400_TRF_ time lags are shown in Figure 1D. In the natural condition, N1_TRF_ and P2_TRF_ components dominated the central region, peaking at electrode FC4 [t(49) = +7.49, *p* < 0.05, FDR-corrected] and electrode C3 [t(49) = +12.10, *p* < 0.05, FDR-corrected], respectively. In contrast, the vocoded condition exhibited dominance in the P400_TRF_ component, peaking at electrode Cz [t(49) = −7.49, *p* < 0.05, FDR-corrected]. Across all 64 channels, *t*-values ranged from −7.49 to +12.10 (Figure 1D).

### 3.2 Control analysis: alpha power as a marker of alertness

**Figure 2:**
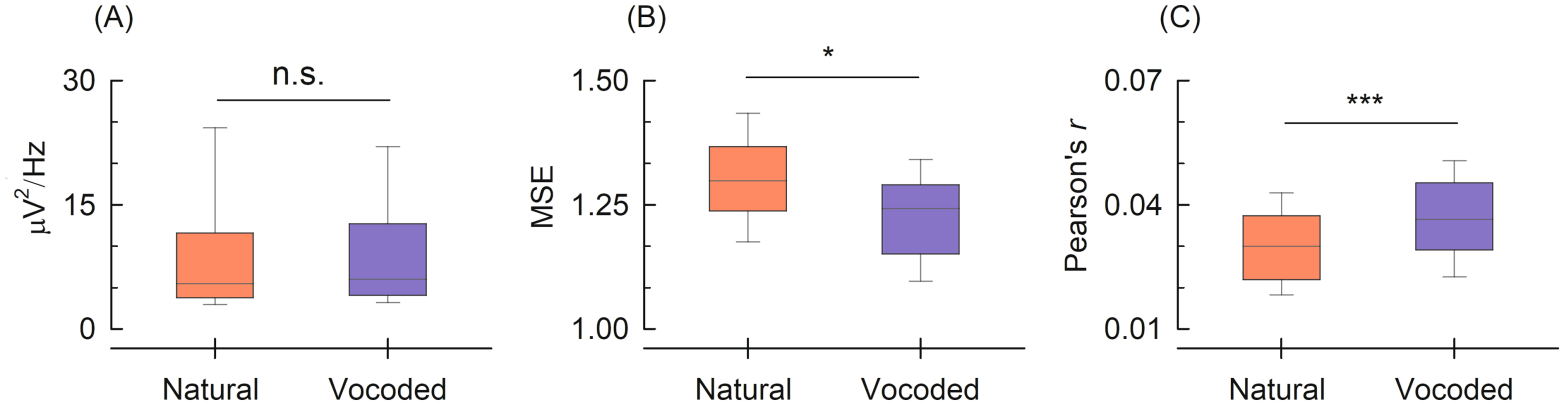
**(A)** Alpha power (8–12 Hz) across conditions. **(B)** Mean squared error (MSE) of TRF model prediction. **(C)** Pearson correlation between stimulus envelope and neural response. Box plots display median (center line), interquartile range (box), and full range (whiskers) for natural (red) and vocoded (blue) conditions. Asterisks denote statistical significance: *p < 0.05, ***p < 0.001; n.s., not significant (*p* > 0.05).

To address the potential confound of fixed block order, we conducted control analyses examining alpha power (8–12 Hz) of the TRF model across conditions (Figure 2A). Alpha power did not differ significantly between natural (mean: 9.69 μV^2^/Hz, standard deviation (SD): 9.45) and vocoded (mean: 9.50 μV^2^/Hz, SD: 7.94) condition [t(49) = 0.32, *p* = 0.75]. The absence of significant differences indicates that condition effects are not confounded by fatigue or alertness changes.

### 3.3 TRF model performance

To characterize acoustic-neural coupling, we examined measurements of TRF model prediction performance: MSE and Pearson correlation (Figures 2B,C). MSE of the TRF model differed significantly between conditions [Natural: 1.297 ± 0.121, Vocoded: 1.227 ± 0.095, t(49) = 2.51, *p* = 0.016]. Pearson correlation between stimulus envelope and neural response was significantly higher in the vocoded condition compared to the natural condition [Natural: 0.030 ± 0.009, Vocoded: 0.037 ± 0.011, t(49) = −6.43, *p* < 0.001]. Notably, across both natural and vocoded conditions, valid TRFs showed markedly better prediction performance than shuffled TRFs at the subject level for both mean squared error and Pearson correlation (all *p* < 0.001; Table 2), indicating that the reported model performance cannot be explained by chance-level correlations.

**Table 2:** TRF model prediction performance for valid and shuffled models in natural and vocoded speech conditions.

Condition / metric	Valid	Shuffled	t(49)	*p*-value
Natural / r	0.030 ± 0.009	0.007 ± 0.006	21.75	<0.001
Natural / MSE	1.297 ± 0.121	1.299 ± 0.122	−12.95	<0.001
Vocoded / r	0.037 ± 0.011	0.008 ± 0.006	22.12	<0.001
Vocoded / MSE	1.227 ± 0.095	1.229 ± 0.095	−12.60	<0.001

Entries show subject-level Pearson correlation and mean squared error (mean ± SD).

### 3.4 TRF components validation against chance level

**Figure 3:**
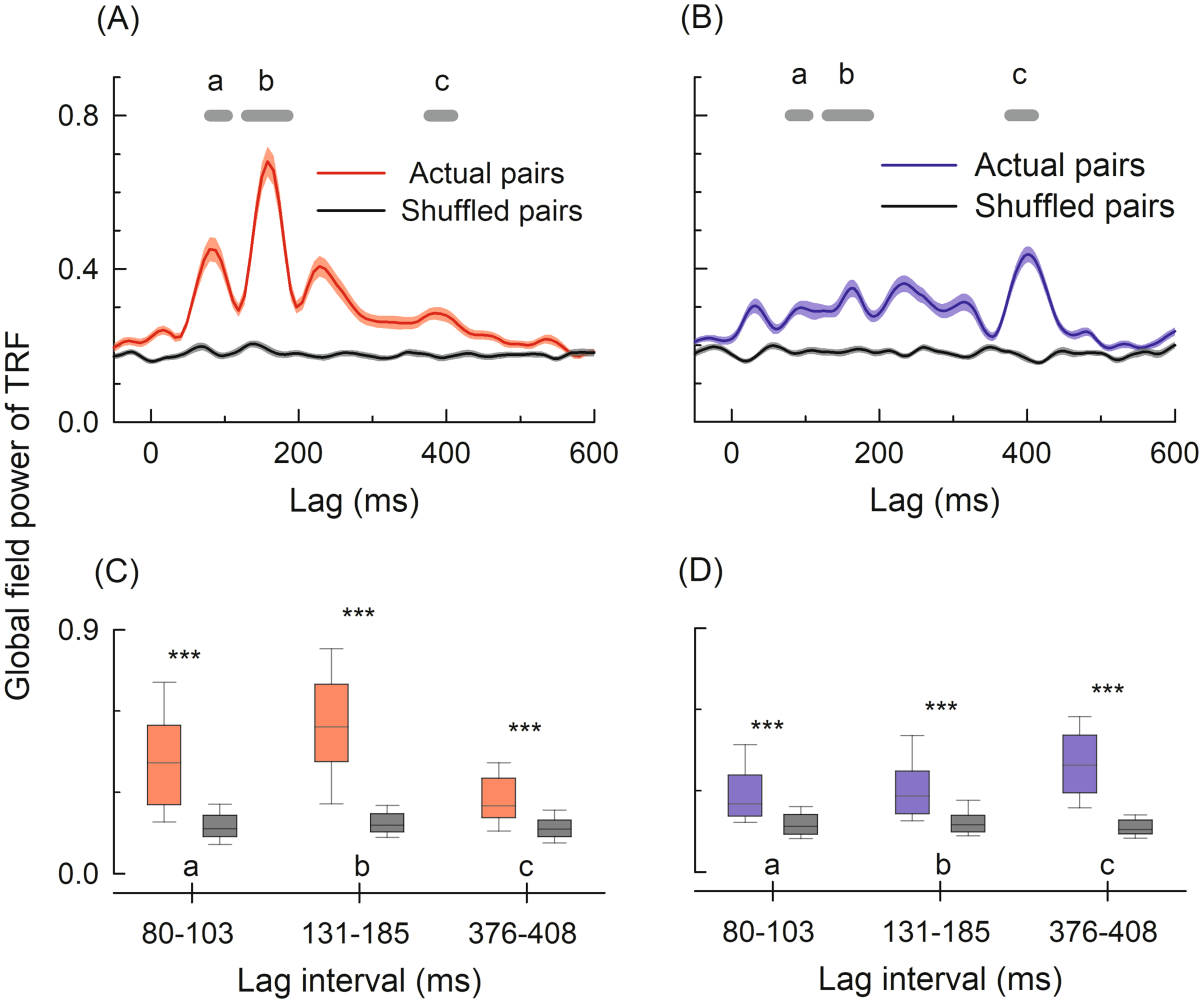
**(A,B)** Grand-averaged TRF waveforms for natural (**A**, red) and vocoded (**B**, blue) conditions, comparing actual stimulus–response pairs (colored lines with shaded 95% confidence interval) against shuffled pairs (black lines). Horizontal gray bars denote the three time windows analyzed (a: 80–103 ms, b: 131–185 ms, c: 376–408 ms). **(C,D)** Global field power (GFP) of valid TRFs (colored) versus shuffled TRFs (gray) for natural **(C)** and vocoded **(D)** conditions within each time window. Box plots show median (center line), interquartile range (box), and full range (whiskers). Asterisks denote statistical significance: *** *p* < 0.001.

Valid TRFs showed significantly higher amplitudes than shuffled TRFs at all three identified components in both conditions (all *p* < 0.001) (Figure 3). For the N1_TRF_ component, valid TRF amplitudes significantly exceeded shuffled controls in both the vocoded condition [Valid: 0.29 ± 0.11, Shuffled: 0.18 ± 0.05, t(49) = 6.29, *p* < 0.001] and the natural condition [Valid: 0.43 ± 0.20, Shuffled: 0.18 ± 0.06, t(49) = 8.44, *p* < 0.001]. Similarly, P2_TRF_ component amplitudes were significantly higher for valid TRFs compared to shuffled TRFs in the vocoded condition [Valid: 0.31 ± 0.11, Shuffled: 0.19 ± 0.05, t(49) = 7.71, *p* < 0.001] and the natural condition [Valid: 0.55 ± 0.21, Shuffled: 0.19 ± 0.05, t(49) = 12.01, *p* < 0.001]. Most notably, the P400_TRF_ component—the late response hypothesized to reflect compensatory processing for degraded speech—showed a robust validation pattern. P400_TRF_ amplitudes significantly exceeded shuffled controls in the vocoded condition [Valid: 0.40 ± 0.13, Shuffled: 0.17 ± 0.05, t(49) = 13.30, *p* < 0.001] and in the natural condition [Valid: 0.28 ± 0.10, Shuffled: 0.17 ± 0.05, t(49) = 6.92, *p* < 0.001]. This robust validation across all three components—particularly the pronounced P400_TRF_ in the vocoded condition—demonstrates that observed TRF amplitudes reflect genuine stimulus–response coupling rather than noise or chance-level correlations. The significantly higher valid TRF amplitudes across all time windows provide definitive evidence that the identified neural responses represent robust, stimulus-locked neural processes underlying intelligibility-driven speech processing.

### 3.5 TRF at source level

**Figure 4:**
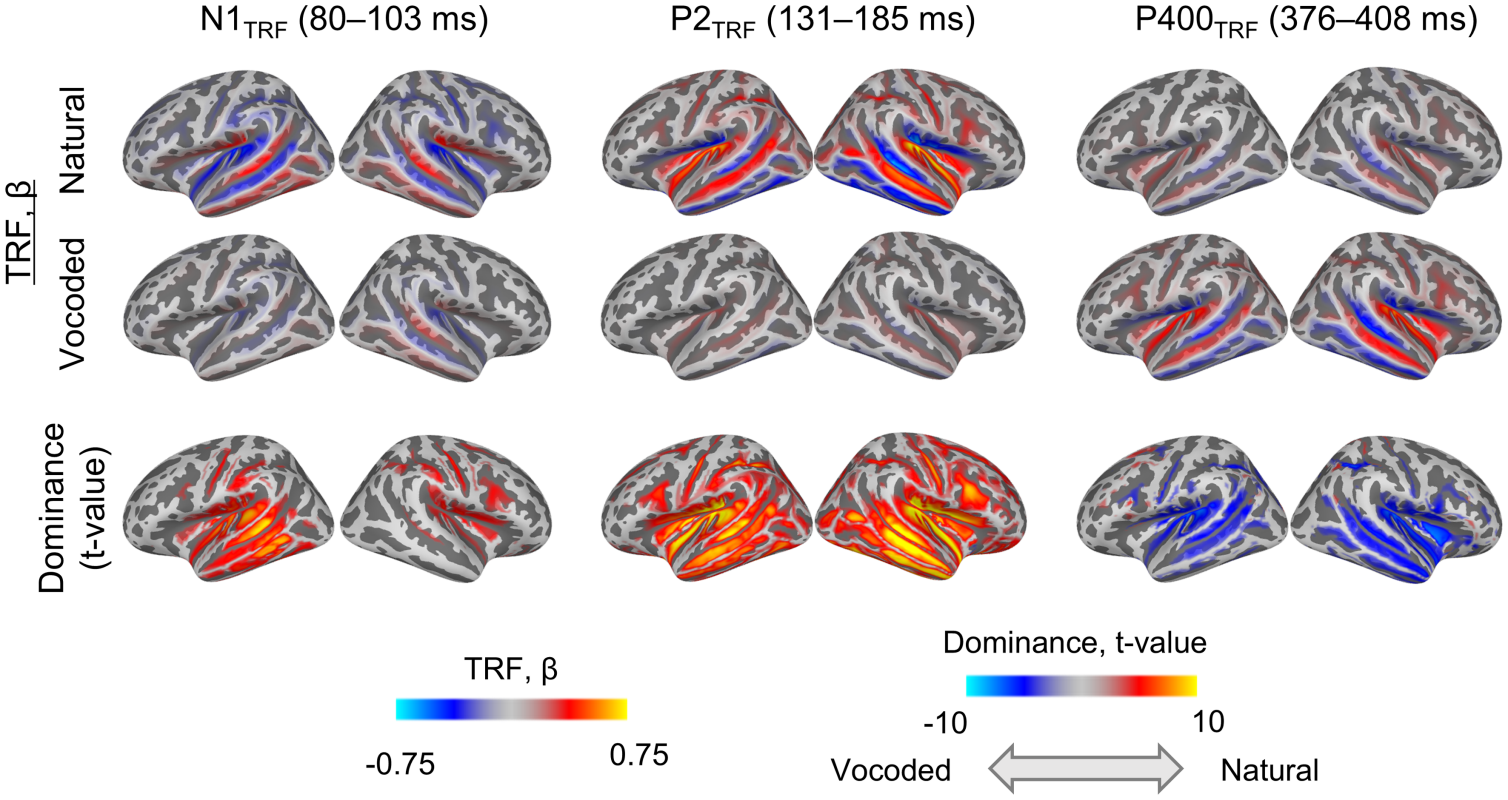
Source localization of the continuous speech-evoked potential at N1TRF (80–103 ms), P2TRF (131–185 ms), and P400TRF (376–408 ms) time lags. The dominance row denotes the *T*-value from paired *t*-tests at each of the 20,484 cortical voxels (blue: vocoded condition > natural condition, red: natural condition > vocoded condition), comparing natural vs. vocoded conditions (*t*_49_, *p* < 0.05, FDR-corrected across all voxels). The mask was applied to display only voxels reaching statistical significance after multiple comparison correction. A mask was applied (*p* < 0.05, FDR-corrected). TRF, temporal response function; FDR, false discovery rate.

At the source level, the mTRF analysis was performed for the N1_TRF_, P2_TRF_, and P400_TRF_ periods, investigating source localization related to speech intelligibility (Figure 4). The natural condition exhibited significant early dominance for N1_TRF_, with the peak voxel located in the left MTG [t(49) = 8.67, *p* < 0.05], and for P2_TRF_, peaking in the left SMG [t(49) = 13.67, *p* < 0.05]. In contrast, the vocoded condition showed dominant P400_TRF_ responses, with the peak voxel located in the right IFG [t(49) = −7.24, *p* < 0.05]. Figure 4 also shows the dominance of both natural and vocoded conditions, with the color bar representing *t*-values from −10 to 10 (paired t-test, FDR-corrected). As observed in Figure 4, the natural condition exhibited dominance in the early components (N1_TRF_ and P2_TRF_), while the vocoded condition showed dominance in the late component (P400_TRF_).

The ratios of significant voxels within 6 ROIs to the condition were calculated (Table 3). For all ROIs, the early components (N1_TRF_ and P2_TRF_) were dominant to the natural condition, while the late component (P400_TRF_) was dominant to the vocoded condition.

**Table 3:** The percentage of significantly dominant voxels in 6 regions of interest (ROIs) related to language processing for N1_TRF_, P2_TRF_, and P400_TRF_ (paired *t*-test, *p* < 0.05, false discovery rate-corrected).

Anatomical region	Dominance (voxel, %)					
	N1_TRF_		P2_TRF_		P400_TRF_	
	Natural	Vocoded	Natural	Vocoded	Natural	Vocoded
IFG	20.42	—	50.00	0.52	8.90	19.63
SMG	36.06	0.39	49.71	—	—	14.23
HG	62.03	5.06	79.75	—	—	70.89
PP of STG	33.01	—	70.87	—	—	51.46
TP of STG	44.87	—	73.72	—	—	54.49
MTG	35.94	—	83.85	—	0.26	61.72

HG, Heschl’s gyrus; IFG, inferior frontal gyrus; PP, planum polare; MTG, middle temporal gyrus; TP, temporal plane; SMG, supramarginal gyrus; STG, superior temporal gyrus.

The proportion of voxels within each ROI showed significant dominance either in the natural or vocoded condition for each TRF component. The early components (N1_TRF_ and P2_TRF_) exhibited higher percentages of significantly dominant voxels in the natural condition, while the late component (P400_TRF_) showed higher percentages in the vocoded condition.

## 4 Discussion

The present study sought to elucidate the time course of degraded speech perception by examining the TRF in response to speech with varying intelligibility. By comparing clear and vocoded speech conditions, we identified distinct neural patterns associated with each condition, offering insights into how the brain manages lexical competition in diverse auditory environments.

Numerous studies have demonstrated that language processing can occur automatically, even when listeners are not actively attending to speech. Neural responses associated with lexical and semantic processing—such as the N400—have been observed in passive listening paradigms, with recent evidence demonstrating that semantic-level markers including N400 and semantic TRFs can be elicited in such contexts (Brodbeck et al., 2018; Yang et al., 2024). Recent work using TRF and continuous speech paradigms further shows that robust neural tracking of linguistic features is present during passive listening, suggesting that core aspects of language comprehension operate automatically and do not require focused attention (Crosse et al., 2016; Brodbeck et al., 2018; Broderick et al., 2018). Importantly, clinical and neuroimaging research confirms that passive auditory paradigms can reliably elicit language lateralization and language-related brain activity (Okahara et al., 2024). In addition, electrophysiological studies have shown that native language advantages in speech processing are evident even in passive listening, supporting the notion that language processing can proceed without active engagement (Yang et al., 2024). Collectively, these findings support the view that passive listening is sufficient to engage the neural mechanisms underlying language processing, allowing researchers to investigate speech comprehension in more naturalistic and ecologically valid settings.

Within a predictive coding framework (Friston, 2005), our findings demonstrate that accurate sensory predictions in clear speech reduce early TRF amplitudes, while increased prediction errors under degraded conditions lead to delayed, amplified late TRF components (P400_TRF_). Our findings revealed that early TRF components, specifically N1_TRF_ and P2_TRF_, exhibited stronger responses in the clear speech condition, indicating more effective initial auditory processing when the speech signal is intelligible (Näätänen, 2001; Krumbholz et al., 2003). This aligns with the predictive coding framework, where accurate top-down predictions facilitate rapid and efficient early sensory processing, minimizing prediction error and supporting fluent speech comprehension (Friston, 2005; Auksztulewicz and Friston, 2016; Sohoglu and Davis, 2016; Heilbron et al., 2022). In contrast, the P400_TRF_ component was delayed and more pronounced in response to degraded speech. Within the predictive coding framework, this late component is interpreted as a neural signature of increased prediction error and the recruitment of higher-order cortical regions to resolve uncertainty (Garrido et al., 2009b; Blank and Davis, 2016; Sohoglu and Davis, 2016; Heilbron et al., 2022). Although this response showed a positive deflection—unlike the canonical N400 observed in traditional ERP studies—such polarity differences are less critical in the context of TRF analyses. TRF analysis estimates the linear stimulus–response mapping (Crosse et al., 2016). As highlighted in previous TRF studies, the timing and functional context of neural responses are more informative than their polarity, which can vary based on analysis methods and referencing schemes (Di Liberto et al., 2015; Crosse et al., 2016; Brodbeck et al., 2018; Broderick et al., 2018). Thus, our key finding is the delayed temporal dynamics of the response, likely reflecting compensatory or additional semantic integration processes as the brain attempts to maintain comprehension when early acoustic features are degraded.

While our paradigm involved passive listening, converging evidence from EEG, MEG, and fMRI studies demonstrates that key markers of linguistic processing—such as the N400 and semantic TRFs—can be elicited even in the absence of explicit tasks (Wacongne et al., 2011; Brodbeck et al., 2018; Heilbron and Chait, 2018; Yang et al., 2024). The late TRF component (P400_TRF_) observed in our study is consistent in timing and cortical localization with these established markers of semantic and lexical processing. Although passive listening may not engage all aspects of conscious language comprehension, these findings support the interpretation that our results reflect genuine linguistic processing, not merely low-level auditory prediction.

An interesting dissociation emerged in measurements of TRF model performance between vocoded and natural conditions. MSE of the TRF model was significantly lower for vocoded conditions (vocoded: 1.227 ± 0.095 vs. natural: 1.297 ± 0.121, *p* = 0.016). Pearson correlation was significantly higher for vocoded speech (vocoded: 0.037 ± 0.011 vs. natural: 0.030 ± 0.009, *p* < 0.001). This dissociation likely reflects differences in neural processing complexity, consistent with recent findings on speech intelligibility (Karunathilake et al., 2023b). Vocoded speech with reduced acoustic variability and more consistent spectral structure constrains neural processing to envelope-driven mechanisms, resulting in more stimulus-locked and thus more predictable neural responses (lower MSE and higher correlation), while natural speech enables complex linguistic and semantic processing beyond envelope tracking, introducing variability that reduces correlation and increases MSE. Within a predictive coding framework (Sohoglu and Davis, 2016), degraded speech simplifies processing to lower-level sensory predictions relying on prediction error minimization (Blank and Davis, 2016), whereas intact speech engages richer, higher-order mechanisms that support the observed P400_TRF_ response as a compensatory error-correction process. An important consideration is whether the reduced P400_TRF_ in the natural condition reflects intelligibility-driven processing or instead arises from repetition suppression and stimulus familiarity effects, given that identical sentence content was presented in both vocoded and natural condition blocks. The component-specific pattern observed in our data argues against a pure familiarity account. Repetition suppression typically manifests as global neural suppression across all components; our findings instead reveal selective enhancement of early components (N1_TRF_, P2_TRF_) coupled with suppression only of the late P400_TRF_ component (Grill-Spector et al., 2006; Garrido et al., 2009a). This selective dissociation is inconsistent with global suppression predicted by repetition suppression or familiarity-driven accounts. Furthermore, alpha power did not differ significantly between blocks [t(49) = 0.32, *p* = 0.75], indicating that systematic changes in neural vigilance or fatigue do not confound the observed condition differences (Engell and Mccarthy, 2014). The source localization of the P400_TRF_ to the inferior frontal gyrus (IFG)—a region consistently associated with semantic processing, error correction, and prediction error detection during language comprehension rather than novelty detection—further supports an intelligibility-driven interpretation (Thompson-Schill et al., 1997; Hartwigsen et al., 2013; Teghipco et al., 2023). Within the predictive coding framework, the vocoded condition necessitates increased reliance on top-down predictions and error correction, manifested as enhanced P400_TRF_ activity, while the natural condition provides sufficient acoustic cues for rapid word recognition with minimal prediction error, reducing the need for late error-correction mechanisms (Blank and Davis, 2016; Sohoglu and Davis, 2016). The observed dissociation between component enhancement (N1_TRF_, P2_TRF_) and suppression (P400_TRF_), combined with stable neural state and appropriate source localization, is most parsimoniously explained by intelligibility-driven neural processing rather than repetition suppression or familiarity effects.

The observed patterns at both the sensor and source levels highlight the brain’s dynamic adaptation to varying auditory environments. In clear speech conditions, TRF components likely reflect successful top-down predictions that minimize sensory surprise, supporting rapid word recognition. However, under vocoded conditions, the brain appears to compensate by prolonging processing, recruiting additional cortical resources to support comprehension. This adaptive mechanism is particularly relevant for individuals with hearing impairments, such as CI users, who often experience degraded auditory signals similar to those simulated by vocoded speech in this study (Wilson and Dorman, 2008). The neural adaptability observed here suggests that even under less-than-optimal listening conditions, the brain may reallocate resources to maintain comprehension, albeit with increased cognitive effort (Giraud and Truy, 2002; Alain et al., 2018).

The increased P400_TRF_ in the vocoded condition may reflect a broader engagement of cognitive resources that supports semantic processing when acoustic cues are degraded. Evidence from continuous speech TRF analyses indicates that late neural components originating from prefrontal and temporal language regions encode semantic and linguistic information, with enhanced responses during high lexical competition scenarios or when acoustic input is compromised (Kegler et al., 2022). This finding is consistent with studies on aging, which show that older adults often exhibit stronger neural tracking of speech envelopes despite poorer signal-to-noise ratios, likely due to compensatory mechanisms aimed at preserving speech comprehension (Presacco et al., 2016; Duta and Plunkett, 2021; Yip et al., 2021). These results suggest that under degraded conditions, the brain increasingly depends on higher-level predictive and semantic processes to support understanding (Ding and Simon, 2013; Peelle and Wingfield, 2016).

It is noteworthy that we used a passive listening condition where subjects watched a silent movie during auditory stimuli were presented. Such conditions are commonly used to divert participants’ attention while still enabling the study of automatic neural responses related to lexical competition and syntactic processing (Pulvermüller et al., 2008; Kong et al., 2014). These previous findings indicate that speech processing can occur without explicit attention, highlighting the robustness of automatic linguistic and lexical processing mechanisms. Our finding is consistent; both short and long-latency TRF components appeared during passive listening, which may suggest speech processing occurs even in the absence of an explicit task. This finding suggests that passive listening paradigms may be a reliable method for studying degraded speech perception, particularly in clinical populations who may have difficulty sustaining attention during active tasks.

Understanding these neural mechanisms is crucial for improving auditory prostheses and rehabilitation strategies for CI users. Enhanced early neural responses in clear conditions could inform the development of auditory training programs to aim at strengthening early prediction-based processing. Conversely, heightened late responses in degraded conditions suggest the importance of interventions that enhance error monitoring and semantic integration. This could include auditory training focused on top-down prediction, or the use of visual or contextual cues to support comprehension under challenging conditions (Sharma et al., 2002a; Giraud and Lee, 2007).

While the present study provides insights into the neural processing of degraded speech, one notable limitation warrants discussion. The behavioral SI test revealed near-ceiling performance in the natural speech condition (mean = 99.65%, SD = 1.01%), precluding examination of correlations between behavioral SI and neural response measures. Future studies employing populations with greater SI variability—such as older adults, individuals with hearing loss, or cochlear implant users—could reveal informative between-subject associations. Additionally, the present study employed normal-hearing young adults, limiting generalizability to other populations with degraded auditory input. Future research should investigate how these neural mechanisms evolve with experience and training, particularly in auditory-impaired populations. Longitudinal studies could provide valuable insights into the neural plasticity associated with degraded speech processing and word recognition strategies. Additionally, exploring the interplay between cognitive factors such as attention, working memory, and neural responses could further elucidate how the brain manages comprehension in challenging listening conditions (Davis and Johnsrude, 2007; McGettigan et al., 2010). Future studies should include explicit word-onset and lexical surprisal predictors to confirm the semantic interpretation of the P400_TRF_ component (Crosse et al., 2016; Brodbeck et al., 2018; Broderick et al., 2018).

While our findings are well explained by the predictive coding framework, an alternative interpretation is the “wait-and-see” strategy (Norris et al., 2016; McMurray et al., 2017). This approach suggests that listeners may delay lexical decisions and accumulate more acoustic information before committing to a word, especially under degraded conditions (Brodbeck et al., 2018; Broderick et al., 2018). The delayed P400_TRF_ component observed in our study could reflect this adaptive, conservative processing style. Although our passive listening paradigm does not directly test this hypothesis, the presence of a delayed neural response in degraded conditions is consistent with this interpretation. Future studies employing active tasks or explicit decision-making paradigms could further clarify the contribution of “wait-and-see” mechanisms to speech comprehension under degraded listening conditions.

In conclusion, this study advances our understanding of the neural dynamics involved in predictive processing under varying speech intelligibility conditions. The differential engagement of early and late TRF components across clear and vocoded speech conditions underscores the brain’s flexibility in processing spoken language. These findings have significant implications for developing auditory prostheses and highlight the importance of tailored rehabilitation strategies that account for the cognitive demands of different listening environments.

## Data Availability

De-identified raw data supporting the conclusions of this article will be made available from the authors upon reasonable request, in accordance with ethics approval and participant consent.
